# Risk of classical Kaposi sarcoma by plasma levels of Epstein-Barr virus antibodies, sCD26, sCD23 and sCD30

**DOI:** 10.1186/1750-9378-5-18

**Published:** 2010-10-12

**Authors:** Colleen Pelser, Jaap Middeldorp, Sam M Mbulaiteye, Carmela Lauria, Angelo Messina, Enza Viviano, Nino Romano, Francesco Vitale, James J Goedert

**Affiliations:** 1 Infections & Immunoepidemiology Branch, Division of Cancer Epidemiology & Genetics, National Cancer Institute, National Institutes of Health, Rockville, Maryland, USA; 2 Department of Epidemiology and Preventive Medicine, University of Maryland School of Medicine, Baltimore, Maryland, USA; 3 Cyto-Barr, Amsterdam, Netherlands; 4 Department of Pathology, Vrije Universiteit Medical Center, Amsterdam, the Netherlands; 5 Lega Italiana per la Lotta Contro i Tumori-Sez Ragusa, Ragusa, Italy; 6 Dipartimento di Scienze Biomediche, Università degli Studi di Catania, Catania, Italy; 7 Dipartimento di Igiene e Microbiologia 'Giuseppe D'Alessandro', Università degli Studi di Palermo, Palermo, Italy

## Abstract

**Background:**

To clarify the immunological alterations leading to classical Kaposi sarcoma (cKS) among people infected with KS-associated herpesvirus (KSHV).

**Methods:**

In a population-based study of 119 cKS cases, 105 KSHV-seropositive controls, and 155 KSHV-seronegative controls, we quantified plasma soluble cluster of differentiation (sCD) levels and antibodies against Epstein-Barr virus nuclear antigen-1 (anti-EBNA-1) and viral capsid antigen (anti-VCA). Differences between groups in prevalence of low-tertile anti-EBNA-1 and high-tertile anti-VCA were compared by logistic regression. Continuous levels between groups and by presence of cKS co-factors among controls were compared by linear regression and Mann-Whitney-Wilcoxon methods.

**Results:**

Comparisons of cKS cases to seropositive controls and of seropositive to seronegative controls revealed no significant differences. However, controls with known cKS cofactors (male sex, nonsmoking, diabetes and cortisone use) had significantly lower levels of anti-EBNA (*P *= 0.0001 - 0.07) and anti-VCA (*P *= 0.0001 - 0.03). Levels of sCD26 were significantly lower for male and non-smoking controls (*P*_adj _≤ 0.03), and they were marginally lower with older age and cortisone use (*P*_adj _≤ 0.09).

**Conclusions:**

Anti-EBV and sCD26 levels were associated with cofactors for cKS, but they did not differ between cKS cases and matched controls. Novel approaches and broader panels of assays are needed to investigate immunological contributions to cKS.

## Background

Kaposi sarcoma (KS) develops in develops in a minority of people who have been infected with Kaposi sarcoma-associated herpesvirus (KSHV). KS is the predominant malignancy occurring in people with the acquired immunodeficiency syndrome (AIDS), illustrating that cell-mediated immunity modifies the risk KS. However, the abnormalities with AIDS are so varied in type and severity that dissecting those specifically associated with KS is challenging. Studies of people who do not have overt immunologic abnormalities may be more informative, although consistent, unambiguous perturbations have yet to be reported in non-AIDS KS [1-4].

KSHV reactivation with viremia is predictive for KS [5], but study of the underlying immunologic mechanisms is technically difficult and unsettled [6-8]. In lieu of a KSHV-specific approach, generic measures of immunity may be helpful. Loss of immunologic control of the related gammaherpesvirus, Epstein-Barr virus (EBV), results in viral reactivation and is marked by higher antibodies against viral capsid antigen (anti-VCA) and lower antibodies against nuclear antigen-1 (anti-EBNA-1). This EBV serology pattern, as well as altered levels of soluble cluster of differentiation (sCD) markers (sCD26, sCD23 and sCD30), have been associated with immune-mediated clinical conditions [9-14]. The Th1/Th2 cellular immunity paradigm has been the rationale [9-14]. We examined whether these markers were associated with classical KS (cKS) in a population-based study in Sicily.

## Results

We included 119 cKS cases, 105 KSHV seropositive controls, and 155 KSHV seronegative controls (Table 1). Anti-EBNA-1 levels ranged from 0.59 - 9.59, and anti-VCA from 0.87 - 9.47. High-tertile anti-VCA was associated with KSHV seropositivity among controls (Table 2, lower panel), but overall anti-EBV levels were not correlated with anti-KSHV levels (Pearson R ≤ 0.07, *P*≥0.29). Radiation and chemotherapy history in cases was infrequent and unrelated to anti-EBV and sCD levels. Excluding such cases did not substantially alter the results (data not shown).

**Table 1:** Distribution of classical Kaposi sarcoma (cKS) cofactors among current study subjects and among all controls in the parent study, KCC-2

	cKS cases		Seropositive controls		Seronegative controls		All KCC-2 controls	
	N	(%)	n	%	n	%	n	%
Sex								
Male	76	(64%)	72	(69%)	100	(65%)	848	(73%)
Female	43	(36%)	33	(31%)	55	(35%)	306	(27%)
Smoking Status								
Never Former Current	58 51 10	(49%) (43%) (8%)	42 44 19	(40%) (42%) (18%)	70 64 21	(45%) (41%) (14%)	439 519 196	(38%) (45%) (17%)
Diabetes								
Yes No	36 83	(30%) (70%)	14 91	(13%) (87%)	44 111	(28%) (72%)	202 952	(18%) (82%)
Cortisone use*†								
Yes No	42 77	(35%) (65%)	18 87	(17%) (83%)	40 113	(26%) (73%)	293 857	(25%) (74%)
	mean	range	mean	range	mean	range	mean	range
Age	75	49-94	73	39-91	73	38-91	71	32-92

*During the last 10 years

†Percentages may equal less than 100 due to missing data

**Table 2:** Risk of classical Kaposi sarcoma (cKS) or KS-associated herpesvirus (KSHV) seropositivity by Epstein Barr virus (EBV) antibody category

EBV antibody tertile category	Cases N (%) 119	Seropositive controls N (%) 105	**OR**_adj_* (95% CI)	**HR**_adj_** (95% CI)
Low EBNA-1	43 (36%)	34 (32%)	0.97 (0.52 - 1.82)	0.93 (0.45 -1.98)
High VCA	42 (35%)	45 (43%)	0.87 (0.45 - 1.69)	1.10 (0.50 - 2.35)
Low EBNA-1/high VCA	5 (4%)	6 (6%)	0.55 (0.15 - 2.00)	0.53 (0.12 - 2.29)
EBNA-1/VCA category				
Low/High Low/Not high Not low/High Not low/not high	5 (4%) 38 (32%) 37 (31%) 39 (33%)	6 (6%) 28 (27%) 39 (37%) 32 (30%)	0.56 (0.14 - 2.18) 1.08 (0.52 - 2.25) 0.98 (0.46 - 2.09) Reference	0.62 (0.13 - 3.10) 1.20 (0.49 - 2.92) 1.23 (0.54 - 2.84) Reference
				

EBV antibody tertile category	Seropositive controls N (%) 105	Seronegative controls N (%) 155	OR_adj_† (95% CI)	HR_adj_‡ (95% CI)
Low EBNA-1	34 (32%)	52 (34%)	0.98 (0.56 - 1.70)	1.04 (0.60 - 1.80)
High VCA	45 (43%)	42 (27%)	1.90 (1.04 - 3.47)	1.88 (1.04 - 3.40)
Low EBNA-1/high VCA	6 (6%)	6 (4%)	1.53 (0.47 -5.02)	1.00 (0.33 - 3.07)
EBNA-1/VCA category				
Low/High Not low/High Low/Not high Not low/not high	6 (6%) 28 (27%) 39 (37%) 32 (30%)	6 (4%) 46 (30%) 36 (23%) 67 (42%)	2.11 (0.61 - 7.30) 1.18 (0.61 - 2.27) 2.02 (1.00 - 4.07) Reference	1.76 (0.52 - 5.93) 1.43 (0.74 - 2.78) 2.20 (1.14 - 4.24) Reference

* Adjusted odds ratio (OR_adj_) comparing cases to KHSV seropositive controls with logistic regression (adjusted for age group, sex, smoking, diabetes, cortisone use)

** Adjusted hazard ratio (HR_adj_) comparing cases to seropositive controls using conditional logistic regression (adjusted for age group, sex, smoking, diabetes and cortisone, and conditioning on assay batch)

† Adjusted odds ratio (OR_adj_) comparing all tested KSHV seropositive controls to seronegative controls with logistic regression models (adjusted for matching factors: age group, sex, smoking and diabetes).

‡ Adjusted hazard ratio (HR_adj_) comparing matched KSHV seropositive and seronegative controls using conditional logistic regression (conditioning on matched set and assay batch).

Mean [standard deviation (SD)] anti-EBNA-1 levels were 5.39 (SD 1.93) in cases, 5.56 (SD 1.72) in seropositive controls, and 5.25 (SD 1.78) in seronegative controls. Mean anti-VCA levels were 5.53 (SD 1.68) in cases, 5.71 (SD 1.82) in seropositive controls, and 5.40 (SD 1.69) in seronegative controls. Adjusted for age and sex, mean differences between cases and seropositive controls were -0.23 [95% confidence interval (CI): -0.70, 0.24] for anti-EBNA-1 and -0.25 (95% CI: -0.72, 0.22) for anti-VCA. With further adjustment for cKS cofactors, anti-EBV levels were virtually identical (mean difference 0.02 for anti-EBNA-1, 0.03 for anti-VCA). Anti-EBV levels did not confound or modify the associations of the cofactors with cKS (data not presented) [15]. Only 5 cases and 6 seropositive controls had low-EBNA-1/high-VCA, which was associated with a non-significantly lower risk of cKS (Table 2, upper panel).

Comparing KSHV-seropositive versus -seronegative controls, adjusted for age and sex, mean differences were 0.34 (95% CI: -0.11, 0.80) for both anti-EBNA-1 and anti-VCA. With further adjustment for cKS cofactors, mean differences were 0.21 (95% CI: -0.24, 0.66) and 0.18 (95% CI: -0.26, 0.62) for anti-EBNA-1 and anti-VCA, respectively.

In the combined group of controls, levels of anti-EBNA-1 and especially anti-VCA were substantially and significantly lower with three cKS cofactors: male sex, nonsmoking, and diabetes (Table 3). The anti-EBNA-1 and anti-VCA levels also were marginally lower with the fourth cKS cofactor, cortisone use (Table 3).

**Table 3:** Differences in Epstein Barr virus (EBV) antibody levels among controls, by risk factors for classical Kaposi sarcoma

	mean difference* (95% CI)	
Cofactor	EBNA-1	VCA
		
Age, continuous	0.00 (-0.02 to 0.02)	0.02 (0.00 to 0.04)
Sex		
Male Female	-1.21 (-1.78 to -0.64) Reference	-2.14 (-2.67 to -1.62) Reference
Smoking status		
Never Former Current	-0.62 (-1.30 to 0.07) -0.82 (-1.41 to -0.23) Reference	-2.37 (-3.01 to -1.73) -1.09 (-1.64 to -0.54) Reference
Diabetes		
Yes No	-0.82 (-1.29 to -0.34) Reference	-0.84 (-1.29 to -0.40) Reference
Cortisone use in last 10 years		
Yes No	-0.41 (-0.89 to 0.06) Reference	-0.46 (-0.89 to -0.02) Reference
KSHV antibody status		
Positive Negative	0.18 (-0.22 to 0.59) Reference	0.11 (-0.27 to 0.49) Reference

* Mean difference from multivariate linear regression models that included all variables shown.

All sCD26, sCD23 and sCD30 levels were within detectable limits. Medians and interquartile ranges were similar across groups (cKS cases, KSHV seropositives, and seronegatives), and no significant differences were found (*P*≥0.35, data not presented).

Considering cKS cofactors in the control subjects, sCD26 levels were higher in females (median 459 ng/mL vs 416 ng/mL, *P*_adj _= 0.006) and current smokers versus never smokers (median 508 ng/mL vs 425 ng/mL, *P*_adj _= 0.03); and sCD26 levels tended to be lower in cortisone users (median 422 ng/mL vs 446 ng/mL, *P*_adj _= 0.09). Older controls tended to have lower sCD26 (*P*_adj _= 0.06) and higher sCD30 (*P*_adj _= 0.08) levels. Otherwise, cKS cofactors were not associated with sCD levels (*P*_adj _> 0.09, data not presented).

## Discussion

Cases of cKS did not differ from KSHV-seropositive controls in anti-EBV or sCD levels. *A posteriori*, we found that cKS cofactors among controls were significantly associated with low anti-EBNA-1 and especially low anti-VCA. Levels of sCD26 tended to be lower in older controls and cortisone users, and they were significantly lower in never smokers and males.

Our assays have been used as measures of immunity for epidemiologic research, specifically invoking the Th1/Th2 cellular immunity paradigm [9,10,16]. However, *in vitro *data to support their use are sparse. Especially with a single plasma sample, these assays may be too crude to distinguish a chronic condition, such as cKS, with the limited statistical power that we had herein.

Although cases did not differ from controls, our findings on cKS cofactors among controls should be noted, particularly because these cofactors are also associated with Th1/Th2 alteration. Th1 responses are generally lower and Th2 responses generally higher in women, smokers, diabetics, and corticosteroid users [17-22]. Classical KS risk is increased for corticosteroid users and perhaps diabetics, but cKS risk is reduced for women and smokers [15,23]. The effect of smoking on Th1/Th2 cytokine levels may also differ by sex [24]. These complex and even inverse relationships suggest that differences in cKS risk may reflect differences in immunity or inflammation that are captured, but only weakly, by anti-EBV and sCD26 levels.

In summary, anti-EBV and sCD26 levels were associated with cofactors for cKS, but not with cKS risk *per se*. Perhaps Th1/Th2 imbalance is poorly measured by these assays or is unrelated to cKS risk. Further studies of cKS risk with a more comprehensive panel of cytokine and inflammatory markers will be needed to illuminate the abnormalities that contribute to this malignancy.

## Methods

The parent case-control study, including KSHV serostatus definition, has been reported [15]. The current study included all cKS cases, all KSHV-seropositive controls, and a sample of KSHV-seronegative controls with ≥0.5 mL previously unthawed plasma for the assays. Seronegatives were matched to seropositive controls and cases on cKS cofactors (sex, age group, cigarette smoking and diabetes).

EBV enzyme immunoassays were performed as described [25]. Antibody levels were the ratio of each specimen's average optical density (OD_450_) divided by the average OD_450_, plus 2 standard deviations, of four negative controls on each plate. Ratio value is highly correlated with anti-EBV immunofluorescence end-point titer [25]. Commercial kits were used to quantify sCD26, sCD23 and sCD30 [Bender MedSystems GmbH (Vienna, Austria)].

Three sets of comparisons were made: cKS cases versus KSHV-seropositive controls; KSHV-seropositive versus-seronegative controls; and all controls by cKS-related cofactors (including KSHV serostatus). We used linear regression (for anti-EBV levels) or Mann-Whitney-Wilcoxon (for sCD levels) methods. As done previously [8,9], cKS risk was postulated to be highest with low-EBNA-1/high-VCA, defined using tertiles among controls. Odds of cKS was estimated with unconditional logistic regression and with conditional logistic regression, conditioning on assay batch.

## List of abbreviations

AIDS: (Acquired Immunodeficiency Syndrome); CI: (confidence interval); cKS: (classical Kaposi sarcoma); EBNA-1: (Epstein-Barr virus nuclear antigen-1); EBV: (Epstein-Barr virus); HR: (hazard ratio); KS: (Kaposi sarcoma); KSHV: (KS-associated herpesvirus); OR: (odds ratio); sCD: (soluble cluster of differentiation proteins); SD: (standard deviation); Th1/Th2: (T-helper type 1/T-helper type 2 cellular immunity); VCA: (Epstein-Barr virus viral capsid antigen).

## Competing interests

The authors declare that they have no competing interests.

## Authors' contributions

CP designed the study, selected the specimens, selected the sCD markers, analyzed the data, and drafted the manuscript. JM performed the EBV antibody and sCD marker assays. SMM helped to design of the study, interpret the results, and edit the manuscript. CL, AM, NR and FV performed all of the field work, including recruitment of the participants, collection of questionnaire data, collection and initial processing of specimens, and shipment of specimens to the repository. NR also performed the KSHV immunofluorescence assays. JJG helped to design the study, interpret the results, and draft the manuscript. All authors read and approved the final manuscript.
